# Optimizing athletic performance through advanced nutrition strategies: can AI and digital platforms have a role in ultraendurance sports?

**DOI:** 10.5114/biolsport.2024.141063

**Published:** 2024-07-23

**Authors:** Luca Puce, Halil İbrahim Ceylan, Carlo Trompetto, Filippo Cotellessa, Cristina Schenone, Lucio Marinelli, Piotr Zmijewski, Nicola Luigi Bragazzi, Laura Mori

**Affiliations:** 1 Department of Neuroscience, Rehabilitation, Ophthalmology, Genetics, Maternal and Child Health, University of Genoa, Italy; 2 Physical Education and Sports Teaching Department, Faculty of Kazim Karabekir Education, Atatürk University, Erzurum, Turkey; 3 Istituto di Ricovero e Cura a Carattere Scientifico (IRCCS) Ospedale Policlinico San Martino, Genova, Italy; 4 Jozef Pilsudski University of Physical Education in Warsaw Poland

**Keywords:** Nutrition, Athletes, Artificial Intelligence, Nutritional knowledge, Ultra-endurance sports

## Abstract

Nutrition is vital for athletic performance, especially in ultra-endurance sports, which pose unique nutritional challenges. Despite its importance, there exist gaps in the nutrition knowledge among athletes, and emerging digital tools could potentially bridge this gap. The ULTRA-Q, a sports nutrition questionnaire adapted for ultra-endurance athletes, was used to assess the nutritional knowledge of ChatGPT-3.5, ChatGPT-4, Google Bard, and Microsoft Copilot. Their performance was compared with experienced ultra-endurance athletes, registered sports nutritionists and dietitians, and the general population. ChatGPT-4 demonstrated the highest accuracy (93%), followed by Microsoft Copilot (92%), Bard (84%), and ChatGPT-3.5 (83%). The averaged AI model achieved an overall score of 88%, with the highest score in Body Composition (94%) and the lowest in Nutrients (84%). The averaged AI model outperformed the general population by 31% points and ultra-endurance athletes by 20% points in overall knowledge. The AI model exhibited superior knowledge in Fluids, outperforming registered dietitians by 49% points, the general population by 42% points, and ultra-endurance athletes by 32% points. In Body Composition, the AI model surpassed the general population by 31% points and ultraendurance athletes by 24% points. In Supplements, it outperformed registered dietitians by 58% points and the general population by 55% points. Finally, in Nutrients and in Recovery, it outperformed the general population only, by 24% and 29% points, respectively. AI models show high proficiency in sports nutrition knowledge, potentially serving as valuable tools for nutritional education and advice. AI-generated insights could be integrated with expert human judgment for effective athlete performance optimization.

## INTRODUCTION

Nutrition plays a crucial role in the performance and overall health of sports athletes [1]. Proper nutrition is, indeed, essential for optimal athletic prowess, as it provides the energy and nutrients athletes need to train, compete, and recover effectively [2]. Key components include i) adequate energy requirements, with athletes needing sufficient calories to fuel their activities, maintain body weight, and preserve their health and well-being, with the precise caloric intake recommended varying depending on the sports discipline, training intensity, and individual metabolism [3]; ii) macronutrient balance, since a balanced intake of carbohydrates, proteins, and fats is vital for athletes, with carbohydrates being the primary energy source during high-intensity activities, proteins being essential for muscle repair and growth, and fats being important for overall health [4, 5]; iii) hydration as staying hydrated is critical for athletes’ performance and health, with sportsmen needing to replace the fluids lost through sweat during exercise [6]; iv) micronutrients, since vitamins and minerals support various bodily functions, including muscle contraction, bone health, and oxygen transport [7]; and v) timing of nutrient intake as the timing of meals and snacks can impact performance and recovery (for instance, eating carbohydrates before exercise can improve performance, while protein and carbohydrate intake after exercise can aid in recovery) [8].

Nutrition is paramount for athletes participating in ultra-endurance sports, which involve events lasting at least 6 hours, who need to carefully manage their nutrition, focusing particularly on maintaining energy and fluid balance [9]. During such extensive activities, athletes might face substantial nutritional challenges, including an energy deficit of about 7,000 kcal per day: this significant energy imbalance can seriously impact health and performance, leading to the loss of fat and skeletal muscle mass, as seen in events like 24-hour swimming, 6-day cycling, or 17-day running [10]. Athletes also experience substantial fluid loss through sweat, potentially up to 2 liters per hour, especially during prolonged exercise or in hot conditions, which can lead to hypohydration [11]. Excessive fluid intake compared to the amount lost can increase the risk of exercise- associated hyponatremia (EAH) and limb swelling [12]. Effective pre-race nutritional strategies should focus on enhancing fat utilization, by consuming fat-rich foods during the race, along with carbohydrates, electrolytes, and fluids [13]. To minimize EAH risks, athletes are advised to include 10–25 mmol of sodium in their fluid intake and limit it to 300–600 mL per hour during the race [14].

Despite the importance of nutrition in sports, athletic dietary practices often fall short of sports nutrition guidelines [15]. One contributing factor to this could be the level of nutrition knowledge among athletes [16]. Unfortunately, the tools used to measure this knowledge are frequently outdated or lack proper validation, resulting in inconsistent findings regarding athletes’ nutrition knowledge [17, 18]. Partly accounting for this, according to a recently published systematic review, the general level of knowledge about sports nutrition varies widely among athletes, with average general and sports nutrition knowledge scores ranging from 40 ± 12% to 70 ± 9%, and with weak-to-moderate positive correlations between nutrition knowledge and healthy dietary behaviors [18].

The overall athletes’ understanding of nutrition is relatively low, with significant gaps and room for improvement. Some sportsmen may have a deep understanding of nutritional principles, while others may have minimal knowledge. This discrepancy, besides the tool used for the assessment, as previously mentioned, often depends on the resources available, such as access to dietitians or nutritionists [19].

Digital platforms have revolutionized the accessibility of nutritional information and personalized dietary advice, with numerous apps and websites dedicated to sports nutrition, offering meal planning, tracking tools, and educational content [20]. These platforms can be highly beneficial for athletes looking to improve their diet and performance. Ultra-endurance athletes prefer, indeed, to seek nutritional advice from fellow athletes and magazines or websites (73%) rather than from nutrition experts (8%) [21].

The advent of generative AI has further expanded the possibilities in sports nutrition, as AI can analyze vast amounts of data to provide personalized nutrition plans based on an athlete’s specific needs, lifestyle, and goals. In the area of sports training process, the emergence of Al-based fitness applications raises pertinent questions regarding their potential to revolutionize personalized health management [20].

However, the scholarly literature on this topic is scarce, making it challenging to draw definitive conclusions. In one of the few studies relevant to the subject, ChatGPT-3.5 and ChatGPT-4 versions, two OpenAI’s advanced large language models, with enhanced capabilities to understand and generate human-like text, were tasked with designing 12-week resistance training programs for both male and female hypothetical subjects [22]. The two AI models generated satisfactory training prescriptions. On the other hand, these contained a few questionable assertions, requiring further fine-tuning before their application. Also, they were not differentiated based on sex/gender, and exhibited some limitations. For instance, the Al models were not able to make real-time revisions or adjustments to training protocols, according to individual progression and user’s feedback. While generally incorporating the well-established, foundational principles of training (variation, specificity, and progressive overload), there were slight differences between the two AI prototypes in terms of selection, frequency, repetition, and intensity of exercise-related variables, with ChatGPT-4 being able to provide more detailed information than Chat- GPT3.5, and tailoring the programs based on training experience levels. Specifically concerning nutrition and hydration, AI-generated advice was deemed “noteworthy” [22].

Another simulation study assessed the efficacy of ChatGPT-4 to generate 30-day exercise prescriptions for five example patient profiles. They were characterized by different fitness objectives and health conditions encompassing scenarios of diverse underlying co-morbidities of varying severity. Co-morbidities included cardiovascular diseases, musculoskeletal disorders, dysmetabolic conditions, respiratory, and mental issues [23]. The AI-generated programs prioritized safety over effectiveness, generally lacking precision in achieving predetermined goals. Moreover, they failed to account for individual biopsychosocial factors and cope with the complexity of specific health conditions and the influence of medications. Besides this overly conservative approach, the model was unable to monitor an individual’s physiological responses to exercise and adjust the protocol accordingly in real-time. The lack of protocol personalization and lack of preliminary patient assessment represented further shortcomings of the AI prototype.

Taken altogether, these recent findings show the potential of AI models and underscore the need to explore whether these AI-based applications can, indeed, offer novel avenues for tailored health enhancement.

However, the effectiveness and accuracy of AI-generated advice must be rigorously tested and validated. A limitation of the previously mentioned studies is that they focused on only one or two AI models. Currently, there exist different AI prototypes, besides those developed by OpenAI (ChatGPT-3.5 and ChatGPT-4), such as Microsoft Copilot and Google Bard (currently known as Google Gemini). All these models share many foundational elements, being built on the Transformer architecture and trained on diverse datasets, which include a broad range of texts from books and websites. These features allow them to generate and understand human-like text, and excel in natural language processing and conversational AI. However, the prototypes vary in terms of model size and outcomes, including performance, accuracy, and coherence. As such, they can differ in handling and reacting to complex or ambiguous queries and generating contextually relevant responses.

Comparing these models, instead of focusing on a single AI prototype, is essential for several reasons: understanding their strengths and weaknesses helps users select the most suitable tool for their specific needs and can contribute to the advancement of AI technology. By analyzing what each AI model excels at and where it falls short, researchers can identify areas for optimization, improvement, and innovation in future models.

More specifically, it is important to ensure that, in the field of sports nutrition, AI recommendations are based on sound nutritional science and are tailored to individual athletes’ needs. This can be done by testing different AI prototypes using reliable tools. To the best of our knowledge, no one has ever appraised AI’s proficiency in nutritional knowledge in ultra-endurance sports. Therefore, the present study was undertaken to fill in this gap of knowledge utilizing a validated sports nutrition questionnaire and comparing the responses of various AI models with those of different human populations and groups, including qualified sports nutritionists, dietitians, athletes, and the general population. The main aim was to identify the strengths and weaknesses of AI-generated advice relative to human expertise in terms of consistency and accuracy, also seeking to pinpoint specific areas where AI models can excel or fall short in providing nutritional advice, paving the way for potential future applications of AI in sports nutrition.

We hypothesized that the AI models, particularly the more advanced versions like ChatGPT-4, would provide nutrition advice comparable to or even superior to that provided by sports nutritionists and dietitians, especially in terms of accuracy and consistency. Also, we expected significant differences in performance among the AI models, with ChatGPT-4 predicted to outperform ChatGPT-3.5, Google Bard, and Microsoft Copilot due to its more sophisticated algorithms and larger training dataset.

## MATERIALS AND METHODS

### Procedure

A literature search was conducted to retrieve a recently published, comprehensive and reliable tool for assessing nutrition knowledge among ultra-endurance athletes. The ULTRA-Q [21], a sports nutrition questionnaire, specifically adapted for testing nutritional knowledge in the specific ultra-endurance athlete population, was found and deemed suitable for the purposes of the present research. The ULTRA-Q has been, indeed, validated both in terms of construct and content validity, showing satisfactory test-retest reliability (with intraclass correlation coefficients ranging between 0.75 and 0.95).

The questionnaire consists of five sections, each targeting a critical aspect of nutrition essential for athletes involved in ultra-endurance sports: namely, “Nutrients” consisting of 37 items, “Fluid” with 8 items, “Recovery” with 11 items, “Body Composition” with 12 items, and “Supplement” with 8 items.

All items from this questionnaire were extracted and submitted to ChatGPT-3.5, ChatGPT-4, Google Bard, and Microsoft Copilot. The four AI models were queried using zero-shot prompts, which means that the machine learning models were queried without any prior specific training or examples given for the task at hand. In other words, the AI-based tools were asked to perform a task they had not explicitly been trained to do, using only their pre-existing knowledge and capabilities. The prompts used were exactly the items from the ULTRA-Q questionnaire [21].

Responses provided by the AI models were, then, collected and scored according to the scoring instructions of the ULTRA-Q questionnaire [21].

### Statistical analysis

Descriptive statistics of the overall assessments and the scores broken down according to each section for each AI model were carried out. Then, the performances of the four AI models were averaged for statistical comparison with the performances of ultra-endurance athletes (extracted from [21]). More in detail, the scores obtained by the four AI models were compared against those achieved by a sample of experienced ultra-endurance athletes (74 males and 27 females) who had filled out the ULTRA-Q in the validation study of the questionnaire. Their overall nutrition knowledge score was computed at 68 ± 10%, without any notable differences between male and female athletes (67 ± 10% and 71 ± 9%, respectively) or between runners and triathletes (69 ± 10% and 65 ± 9%). The scores were also compared with those achieved by ten registered sports nutritionists, further ten registered dietitians, and thirteen members of the general population. All these scores were extracted from [21] and were compared by conducting an analysis of variance (ANOVA) from summary statistics and using the Tukey Honest Significant Difference (HSD) test for the post-hoc analysis.

All statistical analyses were done using the commercial software “Statistical Package for Social Sciences” (SPSS version 28 for Windows, IBM, Armonk, NY, USA).

## RESULTS

### Comparative Analysis of Performance on the ULTRA-Q Questionnaire among the four Artificial Intelligence Models

Overall, Chat-GPT4 demonstrated the highest accuracy, making only 5 mistakes, resulting in an accuracy rate of 93%, followed by Microsoft Copilot, which made 6 mistakes, with an accuracy rate of 92%. Chat-GPT3.5, on the other hand, made 13 mistakes, which translates to an accuracy rate of 83%. Bard, closely following, made 12 mistakes, achieving an accuracy rate of 84%. This data highlights the progressive improvement in accuracy from Chat-GPT3.5 to Chat-GPT4, with Bard positioned in between these two in terms of performance (Table 1).

**TABLE 1:** Comparative analysis of AI models: a detailed breakdown of accuracy rates in various categories of nutritional knowledge in ultra-endurance sports, highlighting mean performances and variability.

AI Model	Nutrients	Fluid	Recovery	Body Composition	Supplements	Overall
ChatGPT-3.5	28/37 (75.7%)	7/8 (87.5%)	11/11 (100.0%)	10/12 (83.3%)	7/8 (87.5%)	63/76 (82.9%)
ChatGPT-4	34/37 (91.9%)	7/8 (87.5%)	10/11 (90.9%)	12/12 (100.0%)	8/8 (100.0%)	71/76 (93.4%)
Google Bard	31/37 (83.8%)	8/8 (100.0%)	8/11 (72.7%)	11/12 (91.7%)	6/8 (75.0%)	64/76 (84.2%)
Microsoft Copilot	32/37 (86.5%)	7/8 (87.5%)	11/11 (100.0%)	12/12 (100.0%)	8/8 (100.0%)	70/76 (92.1%)

Averaged AI model	31.3±2.2 (84.5±5.9%)	7.3±0.4 (90.6±5.4%)	10.0±1.2 (90.9±11.1%)	11.3±0.8 (93.8±6.9%)	7.3±0.8 (90.6±10.4%)	67.0±3.5 (88.2±4.7%)

Concerning the different sections of the ULTRA-Q questionnaire, ChatGPT-3.5 showed strong performance across most categories, particularly excelling in “Fluids” and “Recovery” with scores above 88% and reaching 100% in “Recovery”. Its weakest area appears to be “Nutrients”, with a 76% score. ChatGPT-4 stood out with exceptionally high scores, surpassing 90% in nearly all categories and achieving perfect scores in “Body Composition” and “Supplements”. Bard demonstrated solid knowledge, particularly in “Fluid” with a perfect score and in “Body Composition” with 92%. However, it showed relative weakness in “Recovery”, scoring 73%. Finally, Copilot also exhibited robust performance, especially notable with perfect scores in “Recovery”, “Body Composition”, and “Supplements”. Like Bard, its lowest score was in “Nutrients”.

**FIG. 1:**
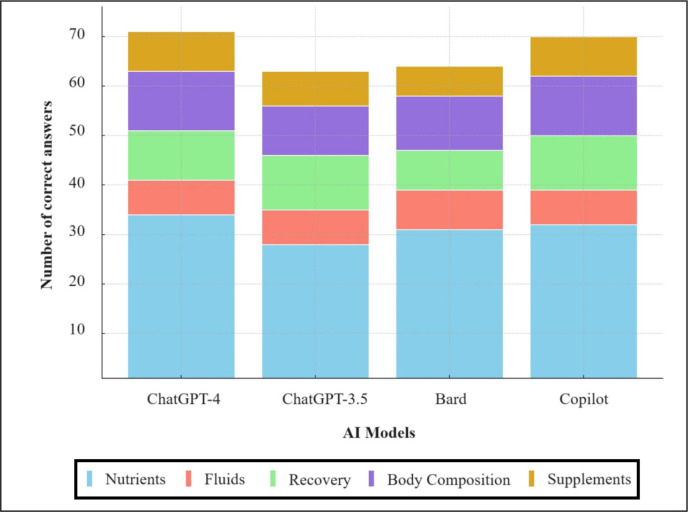
Comparative analysis of AI models in ultra-endurance sports nutrition: a stacked bar chart illustrating the number of correct answers on the ULTRA-Q by ChatGPT-4, ChatGPT-3.5, Bard, and Copilot across key nutrition categories.

The Averaged AI Model, representing the mean performance of these four models, showed consistently high scores across all sections, with the highest in “Body Composition” (94%) and the lowest in “Nutrients” (84%). This model achieved an overall score of 88%, reflecting a strong aggregate capability across various aspects of sports nutrition in ultra-endurance disciplines. This analysis revealed the strengths and weaknesses of each AI model in different nutrition-related areas, with ChatGPT-4 and Copilot generally outperforming others. The Averaged AI Model’s performance indicates that while individual models have their specific areas of expertise, collectively they provide a well-rounded and comprehensive understanding of sports nutrition as reflected by scores achieved on the ULTRA-Q questionnaire (Table 2, Figure 1).

**TABLE 2:** Comparative analysis of knowledge in ultra-endurance sports nutrition: statistical insights from ULTRA-Q questionnaire responses across averaged AI model (AvAI), registered sports nutritionists (SENr), registered dietitians (RD), ultra-endurance athletes (Ultra-End), and general population.

ULTRA-Q Sections	ANOVA	Tukey HSD Post-hoc Test			

		AvAI vs SENr	AvAI vs RD	AvAI vs Ultra-End	AvAI vs GenP
Overall	F = 19.14 (p = 0.0000)	4.1 ([95%CI -10.5 to 18.6], p = 0.9379)	11.9 ([95%CI -2.7 to 26.4], p = 0.1653)	19.9 ([95%CI 7.3 to 32.4], p = 0.0002)	30.8 ([95%CI 16.7 to 44.8], p = 0.0000)

Nutrients	F = 16.80 (p = 0.0000)	-4.4 ([95%CI -21.9 to 13.0], p = 0.9552)	-3.3 ([95%CI -20.8 to 14.1], p = 0.9841)	13.7 ([95%CI -1.4 to 28.7], p = 0.0934)	23.6 ([95%CI 6.7 to 40.4], p = 0.0016)

Fluid	F = 9.61 (p = 0.0000)	14.3 ([95%CI -13.9 to 42.5], p = 0.6265)	49.3 ([95%CI 21.1 to 77.5], p = 0.0000)	32.4 ([95%CI 8.1 to 56.7], p = 0.0030)	41.6 ([95%CI 14.4 to 68.9], p = 0.0004)

Recovery	F = 8.70 (p = 0.0000)	-1.8 ([95%CI -25.9 to 22.3], p = 0.9995)	1.8 ([95%CI -22.3 to 25.9], p = 0.9995)	13.1 ([95%CI -7.7 to 33.9], p = 0.4104)	29.4 ([95%CI 6.1 to 52.7], p = 0.0058)

Body Composition	F = 4.71 (p = 0.0014)	17.1 ([95%CI -8.2 to 42.3], p = 0.3379)	12.1 ([95%CI -13.2 to 37.3], p = 0.6777)	23.7 ([95%CI 1.9 to 45.4], p = 0.0254)	31.0 ([95%CI 6.6 to 55.3], p = 0.0054)

Supplement	F = 4.36 (p = 0.0024)	21.8 ([95%CI -27.6 to 71.3], p = 0.7398)	58.1 ([95%CI 8.7 to 107.6], p = 0.0125)	39.5 ([95%CI -3.1 to 82.1], p = 0.0831)	55.0 ([95%CI 7.2 to 102.8], p = 0.0153)

### Comparative Analysis of Artificial Intelligence Performance on the ULTRA-Q Questionnaire versus Professionals and the General Population.

**FIG. 2:**
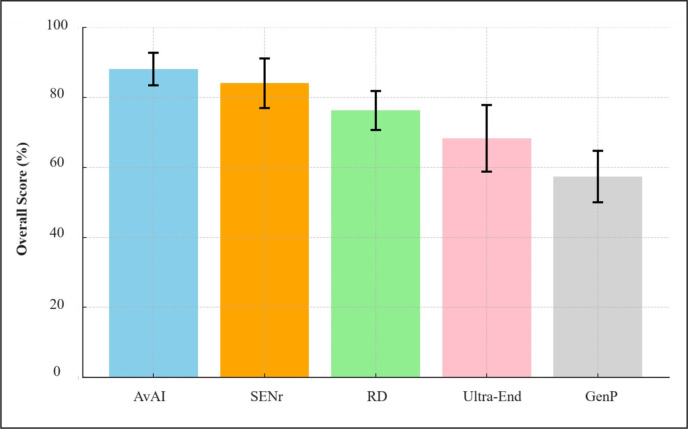
Comprehensive comparison of nutrition knowledge across groups: this chart illustrates the average percentage of correct answers on the ULTRA-Q for AI models, sports and exercise nutrition register (SENr) members, registered dietitians (RD), ultra-endurance athletes, and the general population (GenP). It includes a detailed breakdown by ULTRA-Q categories (Nutrients, Fluids, Recovery, Body Composition, Supplements) and an overall score, providing a holistic view of nutrition knowledge expertise and variability within each group.

In the analysis of the performance of various population groups on the ULTRA-Q, distinct levels of knowledge and consistency could be observed. The averaged AI Model leads with an impressive overall score of 88% and the lowest standard deviation of 5, indicating both high accuracy and consistent responses. Following closely are registered sports nutritionists with a strong performance of 84%, although with a slightly higher standard deviation of 7, suggesting a bit more variability in their responses concerning the averaged AI model. Registered dietitians showed good knowledge with a score of 76% and a standard deviation of 6 (slightly higher than the standard deviation of the Averaged AI model), indicative of moderate consistency in their, expertise. In contrast, ultra-endurance athletes and the general population exhibited lower scores and higher variability in their understanding of sports nutrition. More in detail, ultra-endurance athletes scored 68%, but with the highest standard deviation of 10, reflecting a diverse range of knowledge levels within the group. The general population, as could be expected, scored the lowest at 57% with a standard deviation of 7, highlighting their limited exposure to specialized knowledge in ultra-endurance sports nutrition. This analysis underscores the proficiency and reliability of AI models in providing accurate information, while also emphasizing the varying degrees of specialized knowledge among professionals and the general public (Figure 2).

From a quantitative standpoint, the ANOVA test showed a highly significant difference among groups for the overall scores (F = 19.14, p < 0.0001). The Tukey HSD revealed that the Averaged AI Model significantly outperforms the general population (p < 0.0001) and the ultra-endurance athletes (p = 0.0002), but is not significantly different from the registered sports nutritionists and the registered dietitians. This suggests that while AI models have a notable edge in general knowledge over athletes and the general public, they are on par with professional nutritionists and dietitians. Concerning the section “Nutrients”, again, significant differences are observed among groups (F = 16.80, p < 0.0001), but the averaged AI model did not significantly outperform any group in this category, with the only exception of the general population (p = 0.0016). This indicates a relatively uniform understanding of nutrients across almost all groups, including the AI models and various human professionals. A significant difference in the scores achieved in “Fluids” could be noted (F = 9.61, p < 0.0001), with the averaged AI model notably outperforming not only the ultra-endurance athletes (p = 0.0030) and the general population (p = 0.0004), but also the registered dietitians (p < 0.0001). This highlights a particular strength of AI in fluid-related knowledge, a gap evident in ultra-endurance athletes and the general population, as well as in expert and qualified professionals. While there is a significant overall difference (F = 8.70, p < 0.0001), the post-hoc test indicates no significant difference between the averaged AI model and any other group in the “Recovery” section, with the only exception of the general population (p = 0.0058). This implies a more level playing field in terms of knowledge concerning this specific section. Finally, both “Body Composition” and “Supplement” sections showed statistically significant differences among groups (F = 4.71 and F = 4.36 respectively, with p < 0.005 in both cases). In “Body Composition”, the averaged AI model significantly outperformed both the ultra-endurance athletes (p = 0.0254) and the general population (p = 0.0054), and in “Supplements”, it significantly surpassed both the registered dietitians (p = 0.0125) and the general population (p = 0.0153). For further details, the reader is referred to Table 2.

All these results, taken together, point to specific areas where AI models excel and where human groups, including expert and qualified professionals, might benefit from additional education or resources.

## DISCUSSION

Nutrition is a foundational element for athletic success, and while digital tools and AI offer promising avenues for personalized nutrition advice, it is crucial to ensure that such guidance is accurate, reliable, and based on scientific principles. In particular, nutrition in ultra-endurance sports is a highly specialized topic that requires not only a broad understanding of general nutrition principles but also specific knowledge about the unique physiological demands of ultra-endurance activities. Given this complexity, the high accuracy rate of AI, and in particular of Chat-GPT4 (93%), is particularly impressive, suggesting that AI-based model have a strong capability in handling niche and specialized subjects, likely due to their advanced training and more extensive knowledge base.

Microsoft Copilot, with an accuracy rate of 92%, Chat-GPT3.5, with 83%, and Bard, with 84%, also performed reasonably well, indicating a good grasp of the subject. However, their slightly lower accuracy rates compared to Chat-GPT4 might reflect limitations in dealing with highly specialized topics, which could be due to less comprehensive training data or less cutting-edge algorithms.

The comparative analysis of the AI models – ChatGPT-3.5, Chat- GPT-4, Google Bard, and Microsoft Copilot – revealed notable differences in their performance in replying to the ULTRA-Q questionnaire and, therefore, in their potential to provide nutrition advice for ultraendurance athletes. These differences can be attributed to several factors, including the sophistication of the underlying codes and algorithms, the extent and quality of training data, and the specific design objectives of each model. ChatGPT-4 consistently demonstrated the highest accuracy and reliability among the AI models tested, likely due to its advanced algorithmic improvements in terms of stateof-the-art machine learning techniques and extensive training on a diverse and large dataset [24–26]. This superior performance proves ChatGPT-4’s ability to handle complex and specialized queries effectively, which is crucial for ultra-endurance sports nutrition that requires a nuanced understanding of physiological demands, nutrient timing, and hydration strategies. ChatGPT-3.5, while competent, lagged behind its successor, being capable of providing accurate advice in certain areas, but lacking the comprehensive depth and consistency seen in ChatGPT-4. These findings are in line with the existing scholarly literature [22], which showed that, when comparing these two AI prototypes, ChatGPT-4 was able to provide more detailed and individualized information than ChatGPT3.5.

Google Bard’s performance was mixed, with notable variations across the domains of the questionnaire, indicating that while this AI model can provide accurate advice in some areas, it may not be as reliable in others, possibly due to differences in training data and algorithmic design compared to ChatGPT models. Bard’s integration with Google’s extensive resources might offer advantages in information retrieval and real-time updates, but its performance variability suggests that it may not yet match the specialized focus of models like ChatGPT-4 in sports nutrition. This is in line with studies showing that Google Bard tends to perform worse than ChatGPT-4 [27].

Finally, Microsoft Copilot, primarily designed for coding assistance and strong in specific programming areas [28], surprisingly performed well in this nutrition context. Copilot’s robust performance in these areas could be attributed to its design for precision and its algorithm’s ability to adapt to different contexts. However, Copilot’s low-performance scores in a few categories of the questionnaire indicated some limitations, probably stemming from its primary focus on coding and software development rather than on general knowledge and conversational AI, like the other AI prototypes tested. Despite this, its high scores in other categories highlight its potential versatility and adaptability.

Altogether, these findings suggest that while all AI models have their strengths, ChatGPT-4’s comprehensive improvements make it the most reliable for providing nuanced and accurate nutrition advice for ultra-endurance athletes, highlighting the continual advancements in AI capabilities, especially in understanding and processing complex and specialized information like nutrition in ultra-endurance sports. These findings corroborate results from the literature [22, 23], which compare the performance of AI models, including ChatGPT- 3.5 and ChatGPT-4, in designing training programs and providing nutrition advice. In these studies, it was found that while these AI models could generate satisfactory training prescriptions, they contained some questionable assertions and required further refinement. This aligns with the present study’s findings, where AI models showed varying degrees of accuracy and consistency in different nutrition categories, with ChatGPT-4 generally outperforming the others. This suggests that while AI models are highly promising, there is still room for improvement, particularly in ensuring the advice is tailored and dynamically responsive to individual needs.

For future applications, integrating AI models like ChatGPT-4 into personalized nutrition strategies by combining AI-generated insights with expert human judgment could significantly enhance the precision and effectiveness of high-quality dietary recommendations.

Specifically, when assessing AI against human individuals, including qualified nutritionists, dietitians, ultra-endurance athletes, and the general population, the comparative analysis of the performance data from the ULTRA-Q nutrition questionnaire revealed insightful trends about knowledge levels across these different groups. Leading is the averaged AI Model: its top score of 88% coupled with the lowest standard deviation of 5 not only signifies its superior knowledge base but also showcases remarkable consistency in its responses. This high accuracy and reliability underscore the potential of AI as a valuable resource in specialized fields like sports nutrition. In comparison, human experts like registered sports nutritionists and registered dietitians also demonstrate strong knowledge, albeit with slightly more variability. The high score of the former group reflects their specialized expertise, though the variability indicated by their standard deviation suggests differences in individual experiences or focus areas within sports nutrition. The latter group, with a broader focus on diet and nutrition, shows commendable competence but less specialization in ultra-endurance sports nutrition, as reflected in their scores and standard deviation.

On the other hand, ultra-endurance athletes and the general population present a different picture. ultra-endurance athletes, while directly involved in the field, exhibit a considerable range in their understanding of nutrition, as indicated by their higher standard deviation. This variation might stem from differing levels of interest or access to nutrition education among athletes [29]. According to a systematic review, athletes demonstrated a level of knowledge that was either comparable to or exceeded that of non-athletes. However, their knowledge did not reach the same level as certain comparison groups, such as students specializing in nutrition. The general population’s lower score can be expected, considering their lack of necessity for specialized knowledge in this niche area. The variability here could be attributed to diverse backgrounds and varying degrees of general health awareness [30].

Overall, all these analyses highlight the intersection of specialized knowledge and its application, underscoring the Al-based model’s prowess in providing accurate, reliable information and the importance of targeted education and knowledge dissemination among professionals and athletes in niche fields like ultra-endurance sports nutrition. The differences in scores and standard deviations across these groups also point towards the potential gaps in knowledge that could be addressed through tailored educational programs, especially for athletes and the general public.

### Strengths and limitations

The present study has several notable strengths. Firstly, it demonstrates a high degree of originality and innovation by investigating the use of AI models to assess nutritional knowledge among ultraendurance athletes, a unique and relatively unexplored topic. The research employs a methodologically rigorous approach by utilizing the ULTRA-Q survey, which is specifically designed for this athletic population and has been validated for reliability and accuracy. This survey helps in providing a detailed evaluation of the performance of various AI models across different groups, including athletes, experts, and the general public. Moreover, the study’s comparative analysis is another major strength. By comparing the AI models’ performance against both the general population and certified sports nutritionists and dietitians, the research offers a comprehensive evaluation of AI capabilities in the field of sports nutrition. This analysis not only highlights the potential of AI models to support nutrition education but also emphasizes the importance of integrating AI insights with expert human judgment to enhance nutritional advice and dietetics. However, the study also has certain limitations that need to be acknowledged. One significant limitation is the limited sample size of ultra-endurance athletes, professionals, and individuals from the general population in the comparative groups. Increasing the sample size would enhance the generalizability of the findings. Additionally, the study briefly mentions the AI models’ inability to make real-time adjustments but overlooks other potential challenges in using AI for sports nutrition. These challenges include the models’ handling of individual variability and specific dietary needs in dynamic conditions, which could impact the practical use of AI in real-world sports environments. Furthermore, the AI models’ outputs were not validated by an unbiased external source.

Incorporating external verification would further enhance the credibility of the AI-generated nutritional advice.

### Future Directions

The present analysis, including the part of the comparison of AI versus human individuals, highlights the continuous advancements in AI technology, with each new version showing improved accuracy and performance. In a study assessing the potential of ChatGPT as a reliable source of nutritional advice, it was found that ChatGPT-3.5, when tasked with three hypothetical scenarios encompassing diverse health conditions, could generate various options of meal plans incorporating basic nutrition principles. However, it failed to properly recommend individual macronutrient distribution, and could not effectively deal with underlying health issues and drug interactions. Similarly, ChatGPT-3.5 was unable to set realistic weight loss goals [31].

In our study, Chat-GPT4’s significantly higher accuracy rate compared to its predecessor (ChatGPT-3.5) and other AI prototypes (Microsoft Copilot and Google Bard) indicates substantial improvements in its underlying algorithms and knowledge base. In the future, dietary counseling of athletes could leverage advanced platforms like machine learning and AI to provide reliable, updated resources, as well as more accurate, personalized dietary recommendations. Overall, the integration of AI and advanced digital platforms in sports nutrition promises to enhance the precision and personalization of dietary strategies, counseling, education, and body composition analysis, potentially revolutionizing the field of sports nutrition and athlete performance optimization.

The juxtaposition of AI performance with that of registered nutritionists, dietitians, and ultra-endurance athletes is intriguing, as it contrasts humans with AI’s intellectual or problem-solving capabilities. However, it is important to note that the unique value of specialized human education and expertise is irreplaceable and cannot be understated, highlighting the importance of integrating AI insights with the discerning judgment of experts in the fields of nutrition and dietetics for the most effective outcomes.

Potential risks and ethical considerations also need to be addressed. These include the possibility of over-reliance on AI for nutritional advice without human oversight, the risk of biased or inaccurate information due to limitations in AI training data, and concerns regarding data privacy and security. It is crucial to ensure that AI recommendations are based on sound nutritional science and are tailored to individual athletes’ needs. This can be done by testing different AI prototypes using reliable tools and continuously updating AI algorithms to reflect the latest scientific findings.

## CONCLUSIONS

This study highlighted the significant advancements in AI technology, particularly in the context of sports nutrition for ultra-endurance athletes. By evaluating the performance of four major AI models - ChatGPT-3.5, ChatGPT-4, Google Bard, and Microsoft Copilot - using the ULTRA-Q nutrition questionnaire, the research demonstrated that AI models, especially the advanced ChatGPT-4, can generate accurate and reliable nutritional advice. The comparison between AI models and human groups, including registered sports nutritionists, registered dietitians, ultra-endurance athletes, and the general population, revealed that AI models can match or even surpass human experts in certain areas of nutrition knowledge. Chat- GPT-4, in particular, exhibited the highest accuracy and consistency, outperforming other AI models and showing strong potential in specialized subjects like nutrition for ultra-endurance sports. The study also identified specific strengths and weaknesses of the AI models in various nutrition-related areas. While AI models excelled in categories such as fluid management and recovery, they showed limitations in more nuanced areas like nutrient timing and specific dietary needs for ultra-endurance activities. Overall, the findings suggest that AI models have the potential to enhance personalized nutrition strategies for athletes, leading to improved performance and health outcomes.

On the other hand, while highlighting the significant advancements in AI and its applicability in sports nutrition, the present study reaffirms the indispensable value of specialized human education and expertise. The integration of AI insights with expert human judgment in nutrition and dietetics could provide more effective and personalized dietary recommendations. This study paves the way for future research and developments in the integration of AI into sports nutrition, aiming to optimize athlete performance and overall well-being.
